# Association of Discrimination, Violence, and Resilience with Depressive Symptoms Among Transgender Women in Rio de Janeiro, Brazil: A Cross-Sectional Analysis

**DOI:** 10.1089/trgh.2020.0171

**Published:** 2022-02-14

**Authors:** Paula M. Luz, Emilia M. Jalil, Jessica Castilho, Luciane Velasque, Michelle Ramos, Ana Cristina G. Ferreira, Ana Luisa Ferreira, Erin C. Wilson, Valdilea G. Veloso, Brett D. Thombs, Erica E.M. Moodie, Beatriz Grinsztejn

**Affiliations:** ^1^ Instituto Nacional de Infectologia Evandro Chagas, Fundação Oswaldo Cruz, Rio de Janeiro, Brazil.; ^2^ Vanderbilt University School of Medicine, Nashville, USA.; ^3^ Centro de Ciências Exatas e Tecnologia, Universidade Federal do Estado do Rio de Janeiro, Rio de Janeiro, Brazil.; ^4^ San Francisco Department of Public Health, San Francisco, California, USA.; ^5^ Lady Davis Institute for Medical Research, Jewish General Hospital, Montreal, Canada.; ^6^ Department of Psychiatry, and Biomedical Ethics Unit, McGill University, Montreal, Canada.; ^7^ Department of Epidemiology, Biostatistics and Occupational Health, and Biomedical Ethics Unit, McGill University, Montreal, Canada.; ^8^ Department of Medicine, and Biomedical Ethics Unit, McGill University, Montreal, Canada.; ^9^ Department of Psychology, and and Biomedical Ethics Unit, McGill University, Montreal, Canada.; ^10^ Department of Educational and Counselling Psychology, and Biomedical Ethics Unit, McGill University, Montreal, Canada.; ^11^ Department of Epidemiology, Biostatistics and Occupational Health, Faculty of Medicine and Health Sciences, McGill University, Montreal, Canada.

**Keywords:** depression symptoms, discrimination, resilience, transgender women, violence

## Abstract

Transgender women experience violence and discrimination that lead to stress responses and contribute to poor mental health. In this analysis of baseline data from *Transcendendo*, a trans-specific open cohort in Rio de Janeiro, Brazil, we hypothesized that the experience of discrimination and violence would be associated with depressive symptoms and that resilience could mitigate this association. Results showed that prior experiences with discrimination and sexual and physical violence were associated with depressive symptoms, while resilience was inversely associated with depressive symptoms. Resilience did not moderate nor mediate the strong effects of discrimination and violence on depressive symptoms in adjusted models.

## Introduction

Transgender women are a vulnerable and marginalized population in most parts of the world, including Brazil. Not only do they carry a significant burden of negative health experiences and stressors, but very little is known about their means of coping with such adversity.^1^ The minority stress model outlined by Meyer^2^ and expanded to transgender people^3^ posits that unique stressors related to gender identity cause adverse health outcomes. Stigma and discrimination toward transgender women result in devaluing, labeling, and stereotyping, which manifest in the loss of status, unfair and unjust treatment, social isolation, and violence^4^ in addition to increased vulnerability to diseases such as HIV/AIDS.^5^ Stigma and discrimination also lead to stress responses that contribute to poor mental health.^6^ Rates of depression, anxiety, substance use, and symptoms of trauma and suicidality are disproportionately high among transgender women.^7–11^

In contrast, protective factors, including family, peer, and social support, may increase resilience and offset the mental health effects of such stress. Different from coping, resilience is an individual's ability to successfully adapt or recover despite adversity.^12^ Resilience is healthy development despite growing up in high-risk environments, functioning in adverse environments, and recovering after an adverse event or deprivation.^13^ Transgender people experience extreme adversity, often beginning in early childhood, yet resilience is an understudied phenomena in trans health research.^14^

The objective of this work was to assess the role of resilience in the relationship between past experience of discrimination and violence with depressive symptoms among transgender women living in Rio de Janeiro. We hypothesized that the experience of discrimination or violence would be associated with depressive symptoms and that resilience could mitigate this association. We examined whether measures of psychological resilience mediated or moderated the association between discrimination or violence and depressive symptoms among transgender women in Rio de Janeiro, Brazil.

## Methods

### Participants

This analysis evaluated baseline data from *Transcendendo*, an open clinic-based cohort of transgender women established in 2015 at Evandro Chagas National Institute of Infectious Diseases, Oswaldo Cruz Foundation, Rio de Janeiro, Brazil. An article describing the cohort's aims and procedures has been published.^15^ Briefly, aiming to longitudinally evaluate health outcomes among transgender women, any person receiving services in our clinic who was aged ≥18 years, assigned male sex at birth and who identified as a woman, *travesti*, transsexual woman, or another gender other than man was invited to enroll in the cohort. After informed consent, participants answered face-to-face questionnaires with trained interviewers. The Institutional Review Board at the Evandro Chagas National Institute of Infectious Diseases approved the study. The present analysis is based on all participants enrolling from August 2015 (cohort establishment) to March 2020.

### Variables in the model

#### Sociodemographic and HIV status

Based on previous studies,^16,17^ evaluated variables included age (continuous), self-reported race/skin color (black vs. other), years of formal education (<8 years vs. ≥8 years), and monthly per capita income (defined as ratio of household income by number of household members, dichotomized at the median). Sex work was assessed with possible response options: yes, current; yes, past; and never. HIV status was determined through HIV testing as per cohort procedures.^15^

#### Psychosocial

Based on previous work,^18^ we assessed discrimination with eight items addressing various work/life-related situations where participants could have experienced discrimination as resulting from their trans identity. These eight items addressed difficulty in getting a job, being fired from a job, being displaced from housing or school, difficulty in acquiring health services, verbal violence/mockery, and discrimination by friends or family members. Possible responses included “Yes,” “No,” “I don't know,” and “Prefer not to answer.” A discrimination score was calculated by summing the number of “Yes” responses to each item such that the higher the score, the more the participant experienced discrimination during lifetime (range 0–8).

Physical violence was assessed with the question “Have you ever been physically hurt because of your trans identity,” and possible responses included “Yes,” “No,” “I don't know,” and “Prefer not to answer.” Similarly, sexual violence was assessed with the question “Have you ever been forced to have sexual intercourse against your will?,” and possible responses included “Yes,” “No,” “I don't know,” and “Prefer not to answer.” The two variables were dichotomized as Yes for those who answered Yes and No for all other.

Resilience was measured with the 10-item Conner-Davidson resilience scale, as measured in previous work,^18^ which is one of the most commonly used resilience measures.^19^ Responses were provided on a 5-point Likert scale from 0 (“not true at all”) to 4 (“true nearly all the time”). Two additional response options (“Don't know” and “Prefer not to answer”) were available and coded as zero. Total scores are calculated by summing responses, range 0–40.

Depressive symptoms were measured with the 10-item version of the Center for Epidemiologic Studies Depression Scale (CESD-10) with a 4-point Likert scale from 0 (“rarely or none of the time”) to 3 (“all of the time”). Two additional response options (“Don't know” and “Prefer not to answer”) were available and subsequently coded as zero. Total scores are calculated by summing responses, range 0–30.

### Statistical analysis

**FIG. 1.**
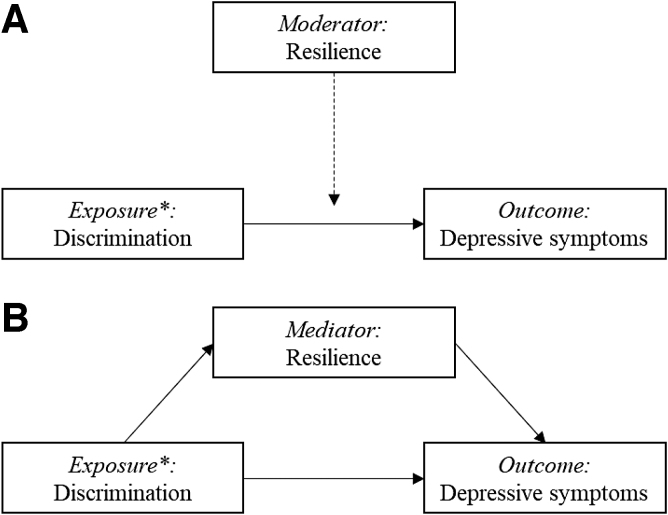
Proposed operational model depicting the relationship between exposures of interest (discrimination, physical violence, and sexual violence) and outcome (depressive symptoms), and the hypothesized mechanisms through which resilience may act. *Exposures of interest include physical and sexual violence, in addition to discrimination.

Variables are described using absolute number and percentages, and means with standard deviations (SDs) or medians with interquartile ranges (IQR), as appropriate. Linear regression models were used to quantify the association of variables with depressive symptom score using a block-wise approach to test if resilience was a potential moderator or mediator. We standardized all continuous explanatory variables (subtracted the mean and divided by SD) to make model estimated effects comparable. The first linear regression model quantified the association of discrimination, physical violence, and sexual violence with depressive symptoms, in addition to age, race, education, sex work, and HIV status. The second model included resilience to assess if it too predicted depressive symptoms and to evaluate if the inclusion of resilience changed the magnitude of the relationship between the predictors and depressive symptom score. The third model tested a two-way interaction term between each exposure of interest (discrimination, physical violence, and sexual violence) and resilience (Fig. 1A). Finally, a mediation analysis was used to test if the association between each exposure (discrimination, physical violence, and sexual violence) and the dependent variable (depressive symptom score) was, in part, mediated by resilience (Fig. 1B). That is, we tested the hypothesis that there would be an indirect effect of discrimination, physical violence, or sexual violence leading to reduced resilience, which would contribute to increased depressive symptom score. R statistical package version 4.0.2 was used for these analyses; packages used included “rms” and “mediation.” We used the mediate function to estimate the average causal mediation effects (indirect effect), average direct effects, and the total effect.

### Ethics statement

The *Transcendendo* cohort and its procedures were reviewed and approved by the Evandro Chagas National Institute of Infectious Diseases Ethics Review Board. All participants signed an informed consent form before study procedures, and files have highly restricted access by any personnel. For the present study, all information was deidentified before analysis.

## Results

### Descriptive statistics

Shown in Table 1, the study population for this analysis included 489 transgender women with mean age of 31.9 years (median 30, IQR 24–37); 27% reported black race, 58% had eight or fewer years of formal education, and a minority (29%) reported never engaging in sex work. Of the 489 participants, 52% reported having experienced physical violence and 42% sexual violence in their lifetime. Discrimination was reported by 470 of 489 participants (96%), with an average of 3.1 experiences of discrimination (range 0–8, median 4 [IQR 2–5]). Resilience score ranged from 0 to 40 with a mean of 29 (median 30, IQR 25–35); depressive symptom scores ranged from 0 to 27 with mean 11.0 and median 10 (IQR 6–15).

**Table 1.** Characteristics of the Study Population

	*N*=489
Age (years)	
Mean (SD)	31.9 (10.2)
Race/ethnicity	
Other	358 (73.2)
Black	131 (26.8)
Education (years)	
≤8	283 (57.9)
>8	206 (42.1)
Monthly per capita income (USD)	
≥180	199 (40.7)
<180	210 (42.9)
Missing	80 (16.4)
Sex work/exchange sex for money/services	
No	142 (29.05)
Yes, current	205 (41.9)
Yes, past	142 (29.05)
HIV test result	
Negative	283 (57.8)
Positive	205 (41.9)
Missing	1 (0.2)
Physical violence	
No	234 (47.9)
Yes	255 (52.1)
Sexual violence	
No	282 (57.7)
Yes	207 (42.3)
Discrimination score^a^	
Mean (SD)	3.1 (2.1)
Resilience score^b^	
Mean (SD)	29.3 (7.5)
Depressive symptom score^c^	
Mean (SD)	11.1 (6.2)

^a^ Discrimination score was based on 8 items, no participant responded “I don't know” or “Prefer not to answer” to >2 items. Number of “I don't know” or “Prefer not to answer” responses by item were: 22 for item 01 (“difficulty getting a job”), 24 for item 02 (“being fired from a job”), 3 for item 04 (“difficulty accessing health services”), 1 for items 6, 7 and 8, and 0 for items 3 and 5.

^b^ Resilience score was based on 10 items: 4 participants responded “I don't know” or “Prefer not to answer” to all items and consequently do not have a resilience score, and 4 additional participants responded “I don't know” or “Prefer not to answer” to one item.

^c^ Depression symptom score was based on 10 items: 2 participants responded “I don't know” or “Prefer not to answer” to all items and consequently do not have a depression score, and 3 participants responded “I don't know” or “Prefer not to answer” to one item.

SD, standard deviation.

### Linear regression models

Results from the linear regression models (Table 2) showed that discrimination, physical violence, and sexual violence were associated with increased depressive symptoms. An increase of 1 SD in the discrimination score led to a 1.51 point increase in depressive symptom score (*p*<0.001). Independently, having suffered physical or sexual violence increased depressive symptom score by 1.41 points (*p*=0.02) and 1.18 points (*p*=0.04), respectively. Model 2 included resilience in addition to all variables considered previously and showed that resilience was negatively associated with depressive symptoms such that a 1 SD increase in the resilience score decreased depressive symptom score by 0.89 points (*p*<0.001). Three models were then considered that included an interaction term between resilience and discrimination (model 3a), physical violence (model 3b), and sexual violence (model 3c). All tested interactions were nonsignificant, and the models' adjusted *R*^2^ showed similar fit to the data compared to model 2. Finally, mediation analysis showed that the effect of discrimination, physical violence, or sexual violence on depression was not mediated by resilience (i.e., the indirect effects of resilience as a mediator were nonsignificant: *p*=0.47 as mediator of discrimination, *p*=0.47 as mediator of physical violence, and *p*=0.62 for mediator of sexual violence). The direct effect of these variables remained significant, while the indirect effect through resilience was not.

**Table 2.** Linear Regression Modelling Results for Predictors of Depressive Symptom Score Among Transgender Women in Rio de Janeiro, Brazil

	Model 1^b^		Model 2^b^		Model 3a^b^		Model 3b^b^		Model 3c^b^	
	B (95% CI)	*p*	B (95% CI)	*p*	B (95% CI)	*p*	B (95% CI)	*p*	B (95% CI)	*p*
Age	−0.46 (−1.01–0.08)	0.09	−0.42 (−0.96–0.12)	0.12	−0.4 (−0.94–0.14)	0.14	−0.42 (−0.96–0.12)	0.13	−0.42 (−0.96–0.12)	0.13
Black race	0.77 (−0.43–1.97)	0.21	0.77 (−0.41–1.96)	0.20	0.79 (−0.39–1.97)	0.19	0.77 (−0.41–1.96)	0.20	0.78 (−0.41–1.96)	0.20
<9y education	−0.01 (−1.1–1.08)	0.99	−0.1 (−1.17–0.98)	0.86	−0.08 (−1.16–1)	0.88	−0.09 (−1.17–0.99)	0.86	−0.08 (−1.17–1)	0.88
Sex work: Current (vs. never)	−0.05 (−1.38–1.28)	0.94	−0.2 (−1.53–1.12)	0.76	−0.23 (−1.55–1.1)	0.74	−0.22 (−1.55–1.1)	0.74	−0.19 (−1.52–1.13)	0.78
Sex work: Past (vs. never)	0.6 (−0.81–2.01)	0.40	0.5 (−0.9–1.9)	0.49	0.53 (−0.88–1.93)	0.46	0.49 (−0.91–1.89)	0.50	0.51 (−0.9–1.91)	0.48
HIV-infected	−0.12 (−1.2–0.96)	0.83	−0.06 (−1.13–1)	0.91	−0.11 (−1.17–0.96)	0.85	−0.07 (−1.14–0.99)	0.89	−0.08 (−1.15–1)	0.89
Discrimination	1.51 (0.92–2.1)	0.00	1.5 (0.91–2.09)	0.00	1.51 (0.92–2.1)	0.00	1.5 (0.92–2.09)	0.00	1.5 (0.92–2.09)	0.00
Physical violence	1.41 (0.24–2.58)	0.02	1.38 (0.22–2.54)	0.02	1.38 (0.22–2.54)	0.02	1.38 (0.22–2.54)	0.02	1.38 (0.22–2.54)	0.02
Sexual violence	1.18 (0.03–2.33)	0.04	1.17 (0.03–2.31)	0.04	1.19 (0.05–2.33)	0.04	1.18 (0.04–2.32)	0.04	1.16 (0.02–2.3)	0.05
Resilience			−0.89 (−1.41–0.37)	0.00	−0.97 (−1.51–0.44)	0.00	−0.78 (−1.54–0.01)	0.05	−0.84 (−1.54–0.14)	0.02
Interaction^a^					−0.3 (−0.83–0.23)	0.27	−0.21 (−1.26–0.83)	0.69	−0.13 (−1.19–0.93)	0.81
Adjusted *R*^2^	0.115		0.134		0.135		0.133		0.133	

^a^ Interaction terms differ by model: 3a includes interaction between discrimination and resilience, 3b includes interaction between physical violence and resilience, and 3c includes interaction between sexual violence and resilience.

^b^ Effective sample for regression models was 483 due to missing data: HIV status was missing for 1 participant; resilience score was missing for 4 participants; and depressive symptom score was missing for 2 participants (one of which did not have a resilience score).

B, beta; CI, confidence interval.

## Discussion

In our study population, prior experiences of discrimination and sexual and physical violence were associated with increased depressive symptoms that were not mitigated by resilience. Resilience was inversely associated with depressive symptoms, but neither moderated nor mediated the strong associations of previous discrimination and violence with depression in adjusted models. Future studies should evaluate if this finding holds for a broader population of transgender women in Brazil and other settings.

Our results also show that most of the participants had experienced physical or sexual violence, while discrimination was reported by a staggering 96% of the participants, with most reporting two to six occurrences of discrimination. These results are, unfortunately, not only plausible but likely representative of the experiences of transgender women in Brazil, the leading country in trans murders worldwide.^20^ Data from the U.S. National Transgender Discrimination Survey that reached over 6000 participants from all 50 U.S. states^21^ show similarly disconcerting results. Discrimination toward trans people has several facets such as in families, schools, jobs, and health services and may include multiple and synergistic layers, such as racism, sexism, and transphobia.^22^ These experiences may affect transgender women's mental health, leading to mental distress,^22^ and thus trigger depressive symptoms. Indeed, we found that violence and discrimination were equally, strongly significantly associated with depressive symptoms in our sample of transgender women.

Although some evidence indicates that discriminated groups, including trans people, are at a higher risk of poor mental health, other data suggest that their adverse experiences may lead to increased resilience.^23,24^ In our study, transgender women with a higher resilience score had lower depressive symptom scores, similar to a U.S. study showing how indicators of resilience were negatively associated with psychological distress.^24^ Although we found that resilience was not an effect modifier of the association between discrimination or violence with depressive symptoms nor did it mediate it, our results showed that it acted independently in the adjusted model, decreasing depressive symptom scores. This corroborates previous findings showing that resilience may reduce depressive symptoms.^9,12,25^ The extreme violence and discrimination transgender women face along with the structural impact of multiple intersecting stigmas must be addressed. In addition, resilience may be an important focus for the maintenance of transgender women's mental health and well-being.^12^

As for study limitations, the cross-sectional assessment means that we cannot be certain of the temporal relationship between the studied variables. Thus, future analysis of longitudinal data is needed. Physical and sexual violence were measured with only one item without information on when it occurred (how long ago) and the degree of exposure (once or repeatedly) and therefore should be interpreted with caution. Likewise, although our measure of discrimination assessed its occurrence in multiple settings, it did not take into account the frequency and timing of the exposure. Furthermore, discrimination, resilience, and depression were assessed with multiple items, and the response options “I don't know” and “Prefer not to answer” were coded as zero, which may bias the sum of the score to lower values. Importantly, future studies should confirm the results presented here with nuanced measures of the exposures of interest. In addition, our open cohort is a convenience sample based mostly on peer referral that is linked to a health service which may bias our population to those who may want or need health care (i.e., for provision of hormonal therapy). Recruitment of a vulnerable minority population is always a major challenge with the caveats that the selected participants may not accurately represent the broader population of transgender women thus impacting the study's generalizability. Finally, while this study focused on depressive symptoms, further investigation into whether and how other adverse mental health outcomes (such as anxiety and substance use) in transgender women are affected by resilience are needed.

Our study corroborates the high vulnerability of transgender women to violence, discrimination, and poor mental health. High rates of discrimination co-occur with worrisome mental health conditions, such as depression. Nevertheless, resilience may be an adaptation to adversity that helps transgender women cope with numerous barriers and adverse contexts faced in their daily lives, especially in highly stigmatized countries such as Brazil. While interventions to eliminate discrimination and violence toward transgender women should be the priority, individual and collective approaches to empower transgender women, engage them as a group, and foster social support may enhance resilience and positive coping as has been done with other sexual minority populations.^26^ Such strategies could be developed as health strategies tailored to transgender women.

## Abbreviations Used

CESD-10: Center for Epidemiologic Studies Depression Scale
IQR: interquartile ranges
SD: standard deviation
